# Matched Endoscopic Sleeve Gastroplasty and Laparoscopic Sleeve Gastrectomy Cases: Formative Cohort Study

**DOI:** 10.2196/29713

**Published:** 2022-11-24

**Authors:** Skye Marshall, Graeme G Rich, Felicity Cohen, Asha Soni, Elizabeth Isenring

**Affiliations:** 1 Bond University Nutrition & Dietetics Research Group Faculty of Health Sciences & Medicine Bond University Gold Coast Australia; 2 Research Institute for Future Health Gold Coast Australia; 3 Australian National University Sydney Australia; 4 Weightloss Solutions Australia Varsity Lakes Australia

**Keywords:** endoscopic sleeve gastroplasty, laparoscopic sleeve gastrectomy, obesity, bariatric surgery, interdisciplinary research, cohort study, metabolic surgery, weight loss, comorbidity, body composition, surgery, gastroplasty, endoscopic surgery, gastroenterology, preoperative, postoperative, outcome, body mass index, sex, age, gastrointestinal, prospective

## Abstract

**Background:**

Bariatric weight-loss surgery rates are increasing internationally. Endoscopic sleeve gastroplasty (ESG) is a novel, minimally invasive endoscopic procedure thought to mimic some of the effects of a more common surgery, laparoscopic sleeve gastrectomy (LSG). Patient factors affecting procedural choice are unexplored.

**Objective:**

This formative study aimed to determine the preoperative and early postoperative characteristics of adults matched for age, sex, and BMI who chose ESG versus LSG.

**Methods:**

This prospective cohort study recruited ESG and matched LSG adults in Australia. Preoperative outcomes were medical history, glycemic biomarkers, blood lipids, liver function enzymes, albumin, blood pressure, hepatic steatosis index, the Gastrointestinal Symptom Rating Scale, the Impact of Weight on Quality of Life–Lite questionnaire, and body composition via dual-energy x-ray absorptiometry. Adverse events were recorded preoperatively and up to 2 weeks postoperatively. SPSS was used to test if there were differences between cohorts by comparing means or mean ranks, and binary regression was used to understand how characteristics might predict procedure choice.

**Results:**

A total of 50 (including 25 ESG and 25 LSG) patients were recruited, who were primarily White (45/50, 90%) and female (41/50, 82%) with a mean age of 41.7 (SD 9.4) years. Participants had a mean of 4.0 (SD 2.2) active comorbid conditions, with the most common being nonalcoholic fatty liver disease (38/50, 76%), back pain (32/50, 64%), anxiety or depression (24/50, 48%), and joint pain (23/50, 46%). The LSG cohort had higher hemoglobin A_1c_ (5.3%, SD 0.2%) than the ESG cohort (5%, SD 0.2%; *P*=.008). There was a 2.4 kg/m^2^ difference in median BMI (*P*=.03) between the groups, but fat and fat-free mass had no meaningful differences. Comparing the LSG and ESG groups showed that the LSG group had lower total quality of life (49.5%, SD 10.6% vs 56.6%, SD 12.7%; *P*=.045), lower weight-related self-esteem (10.7%, IQR 3.6%-25% vs 25%, IQR 17.9%-39.3%; *P*=.02), and worse abdominal pain (38.9%, IQR 33.3%-50% vs 53.9%, SD 14.2%, *P*=.01). For every percent improvement in weight-related self-esteem, the odds for selecting ESG increased by 4.4% (95% CI 1.004-1.085; *P*=.03). For every percent worsening in hunger pain, the odds for selecting ESG decreased by 3.3% (95% CI 0.944-0.990; *P*=.004).

**Conclusions:**

There was very little evidence that Australian adults who chose an endoscopic versus surgical sleeve had different rates of comorbidities, body fat percentage, or weight-related quality of life. There was evidence against the test hypothesis, that is, there was evidence suggesting that lower self-esteem predicted choosing a more invasive sleeve (ie, LSG rather than ESG)

**Trial Registration:**

Australia New Zealand Clinical Trials Registry ACTRN12618000337279; https://anzctr.org.au/Trial/Registration/TrialReview.aspx?id=374595

## Introduction

Despite preventative public health policies enacted by governments around the world, the global prevalence of obesity in adults (defined as BMI ≥30 kg/m^2^) has tripled since 1975, leading to an estimated 650 million individuals with obesity in 2016 [1]. Obesity is a principal metabolic risk factor for chronic comorbidities, including type 2 diabetes mellitus, cardiovascular disease, and obstructive sleep apnea [2]. Diet, exercise, and pharmacotherapy have limited long-term efficacy for weight loss; thus, the number of individuals who elect to undergo bariatric surgery, that is, surgical treatment for obesity, is increasing [3,4]. Bariatric surgery has been demonstrated to result in clinically meaningful weight loss over 1 to 5 years, with studies showing the maintenance of health improvements for up to 10 years [5,6]. Studies have also demonstrated that patients with diabetes, hyperlipidemia, hypertension, and obstructive sleep apnea experience improvement and, in some cases, complete resolution of the comorbidity [7,8].

The United States has the highest number of bariatric procedures performed each year; in 2016, there were 216,000 procedures performed [9]. Other countries with high numbers of bariatric procedures are Brazil (97,480 in 2014), France (46,960 in 2014), Argentina (36,668 in 2014), and Australia (21,043 in 2018-2019) [10,11]. Worldwide, 96% of surgeries are performed laparoscopically; the most common form of the surgery in Australia is laparoscopic sleeve gastrectomy (LSG), which comprises 71.5% of all procedures [11,12].

Despite demonstrating good weight loss efficacy, bariatric surgeries such as LSG have relatively high rates of adverse events (10%-17%), postoperative mortality (0.3%), failure (10%-20%), and weight regain (20%-30%) [13-17]. Additionally, many patients are unwilling to undergo an invasive, incisional, resective surgery, precluding them from an otherwise effective treatment. Endoscopic sleeve gastroplasty (ESG), which is minimally invasive and incisionless, is designed to be analogous to traditional LSG and is being increasingly utilized worldwide [18].

ESG is performed utilizing an endoscopic suturing system (Overstitch, Apollo Endosurgery), coupled with a double-channel flexible gastroscope (GIF2T-180 series, Olympus Optical). These instruments are passed orally into the stomach to allow for full-thickness sutures to permanently plicate the gastric lumen into a narrow “sleeve-like” tubular configuration from the incisura angularis to the fundus [18].

Studies investigating the mechanisms of action of ESG suggest that reduction in gastric volume and improved satiety through delayed gastric emptying contribute to decreased caloric intake and weight loss [5,19]. Available studies report a markedly lower postprocedure complication rate compared to traditional surgical procedures [5]. A chart audit found that among patients with similar preoperative BMI, sex, and age, the ESG cohort had lower weight loss at 12 months but also had fewer complications and a shorter hospital stay [20].

Procedure selection is a complex process involving both medical recommendations and patient preferences. It is unknown if patient demographic or medical characteristics beyond weight loss targets and the risk of complications are associated with procedure choice (ie, surgical vs endoscopic sleeve), which may limit the patient-centeredness of care and interpretation of outcomes. A recent Australian study of unmatched ESG and LSG cohorts suggested factors including preprocedural BMI, quality of life, and gastrointestinal symptoms may play a role [21]. The present formative Australian study aimed to determine the preoperative and early postoperative characteristics of adults matched for age, sex, and BMI who chose ESG versus LSG.

## Methods

This is a substudy of a larger prospective cohort study (Universal Trial Number U1111-1216-8678) undertaken and reported according to the Strengthening the Reporting of Observational Studies in Epidemiology (STROBE) checklist [22] and registered prospectively on March 6, 2018, at the Australia New Zealand Clinical Trials Registry (ACTRN12618000337279). The results of the larger study have been published elsewhere [21].

### Ethical Considerations

This study received ethical approval from the Bond University Human Research Ethics Committee (SM02936); written informed consent was obtained from the included patients by the research team.

### Eligibility Criteria, Matched Cases, and Recruitment

Patients were consecutively recruited from a privately funded outpatient medical clinic in Queensland, Australia, that offered both ESG and LSG procedures. Approximately 500 patients presented to the outpatient clinic during the recruitment phase. Adults aged ≥18 years undergoing either an ESG or LSG procedure from June 2018 to May 2019 were eligible to enroll. Patients unwilling to undergo a dual-energy x-ray absorptiometry (DXA) scan at the local study site were excluded.

Procedural selection for ESG or LSG occurred after a medical consultation with the proceduralists (surgeons or gastroenterologists) that considered the merits of the available procedures, the risks and benefits specific to the patient, and the preference of the patient. Eligibility considerations for the procedures can be found in Multimedia Appendix 1, Table S1.

Due to higher case numbers for LSG than ESG, all eligible ESG and LSG patients were recruited, and each ESG patient was then matched against the available pool of LSG patients for age, sex, and BMI. LSG patients who were not matched against an ESG patient were excluded from inferential data analysis. Matching of patients was blinded to their characteristics and outcomes (other than age, sex, and BMI). Usual care conditions are reported in Multimedia Appendix 1.

### Outcomes

The outcomes of this study were demographic characteristics, body composition, blood pressure, glycemic measures, blood lipids, liver function enzymes, albumin, hepatic steatosis, gastrointestinal symptoms, weight-related quality of life, and adverse events. All outcomes were measured preoperatively, except for perioperative characteristics, which were measured on the day of the procedure, and adverse events, which were measured perioperatively and up to 14 days postoperatively.

### Data Collection Tools

Survey-style data collection tools were self-completed by participants, with all other data being recorded from the study site medical progress notes, pathology reports, and consultation letters or letters of referral from the patient’s general practitioner.

#### Participant Demographic and Preoperative Medical Characteristics

Participant characteristics were recorded from medical records and baseline interviews; they included age, sex, diagnoses of obesity-related comorbidities (including, but not limited to, type 2 diabetes mellitus or prediabetes, hypertension, dyslipidemia, obstructive sleep apnea or use of a continuous positive airway pressure device, osteoarthritis, nonalcoholic fatty liver disease, weight-related joint pain, depression or anxiety, gestational diabetes, polycystic ovary syndrome, and gastroesophageal reflux disease), ethnicity (according to the Australian Bureau of Statistics categorizations), and area of residence (rural or metropolitan). Blood pressure was measured by a registered nurse at the patient’s first preoperative appointment at the study site.

#### Preoperative Body Composition

Measurements of weight, height, and body composition (with DXA) were obtained by trained research assistants at the Bond University Institute of Health and Sport (Robina, Queensland, Australia) using calibrated scales (Wedderburn WM204), a wall-mounted stadiometer with high speed counter (Harpenden Model 602VR; Holtain Limited), and the Lunar Prodigy DXA (Encore Version 14.10.022; GE Medical Systems Lunar), respectively. The assessments were made in a rested, fasting state; participants wore minimal, well-fitting clothing and no jewelry, had their hair down, and had a voided bladder. Height was measured with participants standing with their back to the wall, hands by their sides, feet together, and head in the frontal plane. The height of the board was placed after a breath inhalation and recorded. Fat mass, fat-free mass, and bone mineral content were recorded for the total body, trunk, limbs, and android and gynoid regions. Due to larger body sizes, the DXA scan was implemented by scanning the left and right sides of the body in 2 separate scans to simulate a whole-body scan, rather than using estimated limb mass. For those who failed to attend the DXA scanning session, height and weight were recorded from the patients’ medical records, obtained at their first appointment at the study site, and thus comprised data that were measured, but not calibrated or standardized.

#### Preoperative Comorbid Measures

Systolic and diastolic blood pressure (mm Hg); fasting lipid profile, including total cholesterol, low-density lipoprotein (LDL) cholesterol, high-density lipoprotein (HDL) cholesterol, and triglycerides (mmol/L); fasting serum glucose (mmol/L); hemoglobin A_1c_ (HbA_1c_; presented as a percentage); albumin (g/L); aspartate aminotransferase (units/L); and alanine aminotransferase (units/L) were measured routinely prior to the procedure and were obtained from pathology reports. The hepatic steatosis index (HSI) was calculated using pathology, sex, and BMI and coded as no nonalcoholic fatty liver disease (NAFLD) present when the HSI was <30.0 or NAFLD present when the HSI was >36.0 [23].

#### Preoperative Gastrointestinal Symptoms

Gastrointestinal symptoms, including abdominal pain, reflux, diarrhea, indigestion, and constipation, were evaluated with the Gastrointestinal Symptom Rating Scale (GSRS) [24], a non–disease-specific tool commonly used to evaluate gastrointestinal symptoms following bariatric surgery [25-27]. Completed at the time of recruitment, the GSRS is a 15-item questionnaire that asks participants to rate the symptoms they have experienced in the past week on a Likert scale ranging from 1 (no symptoms) to 7 (severe/frequent symptoms). Subcategories of abdominal pain, reflux, indigestion, constipation, and diarrhea were also calculated. Total and subcategory scores were normalized and reported on a scale of 0 (worst symptoms) to 100 (no symptoms).

### Preoperative Weight-Related Quality of Life

Weight-related quality of life was measured via the Impact of Weight on Quality of Life–Lite (IWQOL-Lite) tool [28] at the time of recruitment. The IWQOL-Lite is a 31-item self-reported measure of 5 domains that affect obese individuals: physical function, self-esteem, sexual life, public distress, and work. Each item reflects experiences in the past week, ranging from “never true” to “always true.” The tool provides a score for each of these domains, as well as a total score, each normalized to a scale of 0 (worst quality of life) to 100 (highest quality of life). The tool is frequently used in studies evaluating bariatric surgery [29,30] and has shown strong retest reliability, internal consistency, and correlation with general quality of life measures, including the 36-Item Short Form Health Survey [28].

### Peri- and Postoperative Adverse Events

All adverse events recorded in medical records were noted and categorized as (1) directly related to the procedure, (2) possibly related to the procedure, or (3) not related to the procedure. Events were further categorized as (1) minor, (2) moderate, or (3) severe, according to the National Institutes of Health guidelines [31]. Severe adverse events were those considered life-threatening, that is, those resulting in death, inpatient hospitalization or prolongation of existing hospitalization, persistent or significant disability or reduced capacity, development of short-bowel syndrome, surgical intervention, or otherwise medically significant events. Moderate adverse events were those that were not considered significant but still required intervention by the medical team, such as prescription of antibiotics, analgesics, or antiemetics beyond the standard postoperative inpatient protocol. Minor adverse events were those that required no intervention beyond reassurance or modified lifestyle.

### Statistical Analysis

All variables were assessed based on the null hypothesis, that is, “there is no difference between 2 independent groups”; therefore, the comparative test for each variable was selected based on how the data met test assumptions. Differences between the ESG and matched and unmatched LSG cases were tested using a chi-square analysis for categorical variables or the Fisher exact test if >20% of cells had an expected count <5. To compare continuous variables between groups, an independent *t* test was used. However, if the variable failed the Levene test for homogeneity of variances, the Welch *t* test was selected. If the variable was highly skewed with the same distribution shapes in both groups, the Mann-Whitney *U* test was used to compare medians. Otherwise, if the variable was highly skewed, with different distribution shapes between groups, the Mann-Whitney *U* test was used to compare rank means. Normality was evaluated using the Shapiro-Wilk test and inspection of histograms and normal Q-Q plots.

The 2 Likert scale–based surveys (ie, the GSRS and IWQOL-Lite) were tested for internal consistency with the Cronbach α, and 95% CIs were generated via the intraclass correlation coefficient [32,33]. Outcome variables that were found to be meaningfully different between procedure types were explored using binomial logistic regression to understand their ability to predict procedure choice. Analysis was performed using SPSS (version 27; IBM Corp).

## Results

### Medical, Nutritional, and Procedural Characteristics of Participants

This study recruited 25 ESG and 56 LSG patients. The 25 ESG patients were matched against the available LSG patients, leading to a final sample of 50 patients, including 25 with ESG and 25 with LSG. Of the matched participants, 9 with ESG and 4 with LSG cancelled their procedure. The cancellation rate was similar between the procedures (*P*=.20). There were no meaningful differences in the baseline characteristics of participants who underwent the procedure and those who did not (Multimedia Appendix 1, Table S3). The reasons for procedure cancellation were financial (1 LSG and 2 ESG patients), changing to a noneligible procedure (1 ESG and 1 LSG patient), personal commitments (1 ESG patient), choosing to delay the procedure (1 ESG patient), no longer wanting any procedure (2 LSG patients), or unexplained (4 ESG patients).

The 56 recruited LSG participants differed from the 25 ESG participants in preoperative BMI (ESG: median 33.4, IQR 30.9-36.8 kg/m^2^; LSG: median 39.6, IQR 35.8-44.4 kg/m^2^; *P*<.001), osteoarthritis, HbA_1c_, and HDL cholesterol (Tables 1 and 2). After matching, participants were primarily White (45/50, 90%) and female (41/50, 82%), with a mean age of 41.7 (SD 9.4) years. Participants had a mean of 4.0 (SD 2.2) active comorbid conditions, with the most common being HIS-identified NAFLD (38/50, 76%), back pain (32/50, 64%), anxiety or depression (24/50, 48%), and osteoarthritis or joint pain (23/50, 46%) (Table 1). Participants did not have high rates of cardiovascular or type 2 diabetes risk factors and had normal blood pressure (mean 126/84.8, SD 12.5/9.0 mm Hg), fasting blood glucose (mean 5.2, SD 1.1 mmol/L), LDL cholesterol (mean 3.2, SD 0.7 mmol/L), and triglycerides (mean 1.4, SD 0.8 mmol/L) (Table 2).

**Table 1:** Preoperative characteristics of endoscopic sleeve gastroplasty, matched laparoscopic sleeve gastrectomy, and unmatched laparoscopic sleeve gastrectomy patients.

Characteristics		Unmatched LSG^a^ (N=56)	ESG^b^ (N=25)	Matched LSG (N=25)	Matched LSG vs ESG, *P* value		Unmatched LSG vs ESG, *P* value	
Age (years), mean (SD)		40.4 (8.8)	42.8 (9.9)	40.7 (8.9)	.30		.27	
Female, n (%)		48 (86)	20 (80)	21 (84)	.71		.52	
**Ethnicity, n (%)**						.39		.28
	White	50 (89)	22 (88)	23 (92)				
	Asian	0 (0)	2 (8)	0 (0)				
	Black	1 (2)	0 (0)	1 (4)				
	Indigenous Australian	2 (4)	0 (0)	0 (0)				
	Pacific Islander	1 (2)	0 (0)	0 (0)				
	Not disclosed	2 (4)	1 (4)	1 (4)				
Rural dwelling, n (%)		3 (5)	3 (12)	0 (0)	.24		.29	
Systolic blood pressure (mm Hg), mean (SD) or median (IQR)		120.0 (110.0-131.0)^c^	126.0 (11.4)	121.3 (13.5)	.19		.25	
Diastolic blood pressure (mm Hg), mean (SD) or median (IQR)		82.0 (78.0-90.0)^c^	84.3 (10)	82.7 (10.3)	.57		.70	
Number of comorbidities^d^, mean (SD) or median (IQR)		4.0 (3.0-5.0)^c^	3.7 (2.1)	4.3 (2.3)	.33		.40	
Type 2 diabetes mellitus, n (%)		2 (4)	0 (0)	1 (4)	.50		.48	
Hypertension, n (%)		16 (29)	9 (36)	6 (24)	.36		.50	
Dyslipidemia, n (%)		9 (16)	7 (28)	4 (16)	.25		.21	
Obstructive sleep apnea, n (%)		7 (13)	0 (0)	3 (12)	.24		.07	
Osteoarthritis/joint pain, n (%)		41 (73)	11 (44)	12 (48)	.78		.01	
Nonalcoholic fatty liver disease, n (%)		4 (7)	2 (8)	2 (8)	.70		.61	
Polycystic ovary syndrome^e^, n (%)		9 (18)	2 (10)	3 (15)	.86		.32	
Gastroesophageal reflux disease, n (%)		22 (39)	9 (36)	11 (44)	.56		.78	
Depression or anxiety, n (%)		23 (41)	12 (48)	12 (48)	>.99		.56	
Gestational diabetes mellitus^e^, n (%)		9 (18)	4 (20)	3 (15)	.83		.56	
Impaired fasting glucose, n (%)		1 (2)	0 (0)	0 (0)	N/A^f^		.69	
Back pain, n (%)		41 (73)	15 (60)	17 (68)	.56		.19	
Asthma, n (%)		19 (34)	6 (24)	10 (40)	.23		.37	

^a^LSG: laparoscopic sleeve gastrectomy.

^b^ESG: endoscopic sleeve gastroplasty.

^c^Data presented as median (IQR); other values in this row are mean (SD).

^d^Comorbidities were defined as any currently active chronic disease or syndrome, excluding sporadic conditions (eg, migraines) and allergic symptoms (eg, rhinitis).

^e^Males were excluded from these analyses.

^f^N/A: not applicable.

**Table 2:** Preoperative biochemistry of endoscopic sleeve gastroplasty, matched laparoscopic sleeve gastrectomy, and unmatched laparoscopic sleeve gastrectomy patients^a^.

Preoperative biochemistry	Unmatched LSG^b^ (N=49)	ESG^c^ (N=19)	Matched LSG (N=21)	Matched LSG vs ESG, *P* value	Unmatched LSG vs ESG, *P* value
Fasting blood glucose (mmol/L), mean (SD) or median (IQR)	5.2 (4.6-5.7)^d^	5.1 (0.4)	4.9 (4.6-5.5)^d^	.30	.77
HbA_1c_ (%), mean (SD) or median (IQR)	5.2 (5.0-5.3)^d^	5.0 (0.2)	5.3 (0.2)	.008	.004
Total cholesterol (mmol/L), mean (SD)	5.2 (0.7)	5.4 (1.1)	5.5 (0.4)	.10	.19
Low-density lipoprotein cholesterol (mmol/L), mean (SD)	3.3 (0.7)	3.2 (0.8)	3.4 (0.5)	.35	.96
High-density lipoprotein cholesterol (mmol/L), mean (SD) or median (IQR)	1.1 (1.0-1.5)^d^	1.5 (0.5)	1.3 (0.3)	.06	.02
Triglycerides (mmol/L), median (IQR)	1.3 (0.9-1.9)	1.1 (0.6-2.2)	1.0 (0.77-1.9)	.76	.62
Alanine aminotransferase (units/L), median (IQR)	28.0 (21.0-46.5)	30.0 (20.0-40.0)	27 (19.5-40.0)	.70	.70
Aspartate aminotransferase (units/L), mean (SD) or median (IQR)	23.0 (18.3-30.8)^d^	28.3 (11.5)	22.5 (16.8-33.8)^d^	.22	.22
Albumin (g/L), mean (SD) or median (IQR)	40.5 (37.3-42.0)^d^	40.0 (20.0-42.0)^d^	40.7 (4.1)	.36	.92
Hepatic steatosis index, mean (SD)	52.6 (6.4)	45.1 (7.4)	47.8 (4.9)	.18	.002
Hepatic steatosis index–derived nonalcoholic fatty liver disease, n (%)	48 (100)	17 (89)	21 (100)	.22	.08

^a^N values differ from Table 1 because preoperative pathology measurements were not available for all participants.

^b^LSG: laparoscopic sleeve gastrectomy.

^c^ESG: endoscopic sleeve gastroplasty.

^d^Data presented as median (IQR); other values in this row are mean (SD).

### Body Composition

Although all recruited patients were booked for a DXA scan, only 29 of 50 (60%) attended (there was no meaningful difference in attendance rate by procedure type; *P*=.15). A total of 3 participants, including 1 with LSG and 2 with ESG, attended the DXA scan 2 to 12 days after commencing a very low-calorie diet (VLCD). DXA scans were completed 3 to 79 days prior to surgery. Despite being matched for age, sex, and BMI, the BMI between groups was different (the difference in medians was 2.4 kg/m^2^; *U*=196.0; *P*=.02; Table 3). Body composition, measured via DXA, showed that LSG participants had higher means or medians for both fat mass and fat-free mass, a clinically meaningful difference. There was a 6.7-kg difference in mean total-body fat mass (*P*=.14) and a 7.7-kg difference in mean total-body fat-free mass (*P*=.11).

**Table 3:** Preoperative body composition of matched endoscopic sleeve gastroplasty and laparoscopic sleeve gastrectomy patients.

Characteristics	Endoscopic sleeve gastroplasty (N=12)	Laparoscopic sleeve gastrectomy (N=17)	*P* value
BMI (kg/m^2^), median (IQR)	33.4 (30.9-36.8)	35.8 (34.9-38.2)	.02
Total-body fat mass (kg), mean (SD)	46.9 (8.7)	53.6 (13.3)	.14
Total-body fat mass^a^ (%), mean (SD)	47.9 (3.5)	50.0 (7.0)	.64
Total-body fat-free mass (kg), median (IQR) or mean (SD)	44.7 (42.9-50.3)^b^	52.4 (9.9)	.11
Total-body fat-free mass^a^ (%), median (IQR) or mean (SD)	49.3 (3.4)	46.5 (43.7-51.0)^b^	.16
Total-body bone mineral content (g), median (IQR) or mean (SD)	2635.5 (2460.5-2787.3)^b^	2856.1 (471.0)	.23
Total-body bone mineral content (%), mean (SD)	2.8 (0.2)	2.6 (0.4)	.20
Android fat mass (kg), mean (SD)	4.5 (1.4)	5.2 (1.4)	.18
Android fat mass^c^ (%), mean (SD)	55.2 (5.0)	56.7 (6.1)	.49
Gynoid fat mass (kg), mean (SD)	8.2 (1.7)	9.1 (2.7)	.33
Gynoid fat mass^c^ (%), median (IQR) or mean (SD)	50.7 (4.5)	52.3 (46.6-55.9)^b^	.31
Android to gynoid fat mass ratio, mean (SD)	0.54 (0.11)	0.60 (0.18)	.36
Android to gynoid fat^c^ ratio, median (IQR) or mean (SD)	1.10 (0.13)	1.10 (1.04-1.27)^b^	.57
Trunk fat mass (kg), mean (SD)	25.1 (5.7)	28.4 (6.7)	.18
Trunk fat mass^c^ (%), mean (SD)	50.4 (3.5)	52.0 (5.6)	.40
Upper limb fat mass (kg), mean (SD)	4.5 (0.6)	5.5 (1.5)	.04
Upper limb fat mass^c^ (%), median (IQR) or mean (SD)	47.8 (5.6)	51.7 (45.8-54.7)^b^	.51
Lower limb fat mass (kg), mean (SD)	16.3 (3.8)	18.8 (6.2)	.20
Lower limb fat mass^c^ (%), median (IQR) or mean (SD)	47.8 (4.2)	49.9 (43.9-55.5)^b^	.23

^a^Percentage of total body mass.

^b^Data presented as median (IQR); other values in this row are mean (SD).

^c^Percentage of total region mass.

### Gastrointestinal Symptoms and Quality of Life

The GSRS (15 items) and IWQOL-Lite (31 items) were completed 2 to 135 days prior to the surgery date, before the VLCD was commenced (except for 1 participant). The Cronbach α for both GSRS and IWQOL-Lite scores showed that they had good and very good internal consistency, respectively (GSRS: α=.848, 95% CI 0.778-0.904; IWQOL-Lite: α=.909, 95% CI 0.862-0.945). The LSG participants reported higher levels of perceived abdominal pain in the previous week than the ESG participants. The abdominal pain subcategory score encompasses being “bothered by stomach ache or pain,” “bothered by hunger pains,” and “bothered by nausea” (Table 4). Both groups reported a median score of 100 for reflux symptoms (indicating no symptoms) and minimal constipation and diarrhea. Total gastrointestinal symptoms did not meaningfully differ between groups. In the previous week, LSG participants reported worse weight-related quality of life (*P*=.045), which appeared to be primarily driven by worse weight-related self-esteem (*P*=.02; Table 4). The groups had similar results in subcategories of weight-related physical function, public distress, and work.

**Table 4:** Preoperative gastrointestinal function and quality of life of matched endoscopic sleeve gastroplasty and laparoscopic sleeve gastrectomy patients.

Scores		Endoscopic sleeve gastroplasty (N=25)	Laparoscopic sleeve gastrectomy (N=25)	*P* value
**Gastrointestinal function scores^a^**				
	Abdominal pain (%), median (IQR) or mean (SD)	53.9 (14.2)	38.9 (33.3-50)^b^	.01
	Reflux (%), median (IQR)	100 (83.3-100)	100 (83.3-100)	.92
	Indigestion (%), mean (SD)	62.9 (20.6)	67.8 (19.4)	.39
	Constipation, (%), median (IQR)	88.9 (55.6-100)	88.9 (77.8-94.4)	.89
	Diarrhea (%), median (IQR)	94.4 (77.8-94.4)	94.4 (72.2-97.2)	.19
	Total gastrointestinal symptom (%), mean (SD)	71.8 (15.4)	71.2 (13.7)	.88
**Quality of life scores^c^**				
	Weight-related physical function (%), mean (SD)	57.5 (17.7)	53.3 (18.1)	.41
	Weight-related self-esteem (%), median (IQR)	25 (17.9-39.3)	10.7 (3.6-25)	.02
	Weight-related sexual life (%), mean (SD)	49.7 (28.3)	42 (20.5)	.28
	Weight-related public distress (%), median (IQR) or mean (SD)	70 (65-95)^b^	67.2 (21.7)	.18
	Weight-related work (%), mean (SD)	68.4 (19.5)	68.5 (16.3)	.99
	Total weight-related quality of life (%), mean (SD)	56.6 (12.7)	49.5 (10.6)	.045

^a^Scores normalized to 0% for worst symptoms and 100% for no symptoms.

^b^Data presented as the median (IQR); other values in this row are mean (SD).

^c^Scores normalized to 0% for worst quality of life and 100% for best quality of life.

### Adverse Events

Two perioperative events were noted. One ESG participant’s procedure was abandoned perioperatively due to the identification of 3 large gastric ulcers. This participant was treated with pantoprazole for 8 weeks and the procedure was then rescheduled and performed. A second participant was scheduled for an ESG; however, the procedure was abandoned perioperatively due to the identification of a large hiatus hernia. This participant was rescheduled and received an LSG from the same proceduralist. There were no procedure-related peri- or postoperative adverse events.

### Procedure Choice

Simple binary logistic regression was performed for factors that had relevant differences in effect size between procedures. For every percent improvement (ie, score increase) in weight-related self-esteem, the odds for selecting ESG increased by 4.4% (odds ratio [OR] 1.044, 95% CI 1.004-1.085; *R*^2^=0.15; *P*=.03). For every percent improvement (ie, score increase) in abdominal pain, the odds for selecting ESG decreased by 7.2% (OR 0.928, 95% CI 0.873-0.987; *R*^2^=0.183; *P*=.02).

Ad-hoc testing was performed to understand how abdominal pain predicted procedure choice. The 3 items contributing to the abdominal pain subcategory (including stomachache, hunger pain, and nausea) were further explored. Stomachache (OR 1.007, *P*=.50) and nausea (OR 1.014, *P*=.31) were found not to be associated with procedure choice, whereas for every percent worsening in hunger pain, the odds of selecting ESG decreased by 3.3% (OR 0.967, 95% CI 0.944-0.990; *R*^2^=0.25; *P*=.004). 

## Discussion

### Principal Findings

This is the first study to explore differences between matched participants who elected to have an LSG or the nonsurgical alternative, ESG. For the participants, who were similar to typical bariatric patients in Australia [11], only weight-related self-esteem and perceived abdominal pain were predictors of procedure choice (ie, surgical vs endoscopic sleeve). However, ad-hoc testing of how abdominal pain predicted procedure choice suggested the relationship was not clinically relevant. In the abdominal pain subgroup, increased pain predicted the choice of LSG, but item analysis found only hunger pain, not stomachache or nausea, predicted the choice of ESG.

Despite being matched for BMI, the rank sums were different between groups. This can be explained by the recruited ESG participants having a lower median BMI than the 56 recruited LSG participants. In this study, the LSG participants that we selected for matching to the ESG patients predominantly had BMIs lower than the median for the whole sample of unmatched LSG participants. This led to an unusual distribution of LSG BMIs, and a higher median and rank sum. This is confirmed by the LSG cohort having a mean fat mass and fat-free mass 6 to 8 kg higher than the ESG cohort. Although problematic in terms of BMI, the body composition of both groups was clinically similar when expressed as a percentage. Both groups presented with high total body fat (48% for ESG and 50% for LSG participants) that was centrally located (the android to gynoid fat percent ratio was 1.10 for both ESG and LSG participants). Importantly, the difference in BMI was not a predictor of procedure choice.

Considering the strong association with obesity of chronic diseases, such as type 2 diabetes and cardiovascular disease, it was surprising that biomarker risk factors and blood pressure measures were normal and there was a low prevalence of impaired fasting glucose, hypertension, type 2 diabetes, and cardiovascular disease. This may be a reflection of the cohort having lower BMIs than typical bariatric candidates (the Australian mean is 41.8 kg/m^2^ [11], compared to the median 33 to 36 kg/m^2^ in this study) and the exclusion of procedures such as Roux-en-Y gastric bypass [34]. Although HbA_1c_ was higher in the LSG participants, this was not clinically relevant, as the difference was small and values were within normal ranges.

### Comparison With Prior Work

The number of cumulative active comorbidities was high (a mean of 4.0 per patient), particularly HSI-identified NAFLD, back pain, and osteoarthritis, which were all present in over 50% of participants. Although the HSI diagnostic criteria for NAFLD identified extremely high rates (90%-100%), only 4 of 50 (8%) patients had a diagnosis in their medical record, aligning with previous research exposing NAFLD underdiagnosis in primary care [35]. The high prevalence of anxiety and depression is also of relevance, highlighting the importance of the psychologist as a core part of the multidisciplinary team. Recent research has found that intensive pre- and postoperative psychological intervention in bariatric surgery patients decreases postoperative symptoms of anxiety and depression compared with standard multidisciplinary care models [36]. The importance of the psychologist as part of the multidisciplinary team is also highlighted by the participants’ extremely low weight-related quality of life in all subcategories compared to nonbariatric surgery population norms for the same BMI category. The most extreme difference was self-esteem, for which population norms for adults with a BMI of 30 to 39.9 kg/m^2^ are 68% to 77%, compared to 11% to 25% in the current sample [37,38]. This is further contrasted against population norms of samples with a BMI of 18.5 to 24.9 kg/m^2^, who have a self-esteem norm of 88% and a total weight-related quality of life norm of 95% [37,38].

### Limitations

No conclusions can be drawn from this study about predictors of procedure choice in participants who were not matched for age, sex, and BMI. Although the preoperative characteristics may be representative of Australian adults who choose an ESG procedure, they are not representative of those who choose an LSG procedure, due to the exclusion of participants who were not matched to ESG candidates. Additionally, there are likely clinically relevant predictors of procedure choice and preoperative differences between ESG and LSG candidates that were not measured in this study, such as fertility, financial situation, and the location of the study site. Under the advice of a statistician, imputation was not used to account for missing data. This decision was made as the authors hypothesized that there would be no differences in outcomes due to being matched, and there were no trends suggesting increased power was required. However, the sample size was small, and although the *P* values when testing outcomes can be considered sufficient evidence against the null hypothesis, they cannot be considered strong evidence. Further research is required to strengthen and confirm our findings. This study was limited by the selection of outcomes, as some outcomes relevant to the sample were not measured, such as fertility, nutrition biochemistry, and bone density. Nevertheless, there were still multiple outcomes measured, and the *P* values were not adjusted (due to lack of power), leading to a chance of type I errors.

### Conclusions

Australian adults who chose an endoscopic or surgical sleeve had high rates of comorbidities, especially NAFLD, back pain, and osteoarthritis, and had high body fat percentage, predominantly centrally located. Most preoperative gastrointestinal symptom scores were low, but abdominal pain was prevalent. Weight-related quality of life was very low compared to weight-adjusted population norms. There was evidence against the test hypothesis, that is, there was evidence suggesting that lower self-esteem predicted choosing a more invasive sleeve (ie, LSG rather than ESG). These preoperative characteristics can be used to improve the patient-centeredness of preoperative and postoperative care and assist in the interpretation of postoperative outcomes between the 2 procedures.

## Appendix

Multimedia Appendix 1 Supplementary materials.

## Abbreviations

DXA: dual-energy x-ray absorptiometry
ESG: endoscopic sleeve gastroplasty
GSRS: Gastrointestinal Symptom Rating Scale
HDL: high-density lipoprotein
HbA_1c_: Hemoglobin A_1c_
HSI: hepatic steatosis index
IWQOL: Impact of Weight on Quality of Life
LDL: low-density lipoprotein
LSG: laparoscopic sleeve gastrectomy
NAFLD: nonalcoholic fatty liver disease
OR: odds ratio
STROBE: Strengthening the Reporting of Observational Studies in Epidemiology
VLCD: very low-calorie diet
