# Supplementary material for: Matched Endoscopic Sleeve Gastroplasty and Laparoscopic Sleeve Gastrectomy Cases: Formative Cohort Study
Source: JMIR Form Res. 2022 Nov 24;6(11):e29713. doi: 10.2196/29713 (PMC9732757; doi:10.2196/29713)
Supplement: Multimedia Appendix 1 [file formative_v6i11e29713_app1.docx]

**Pre-, peri-, and early postoperative characteristics and adverse events of matched adults who elect endoscopic sleeve gastroplasty (ESG) versus laparoscopic sleeve gastrectomy (LSG): A prospective observational study**

Supplementary Materials

# Table S1: Eligibility considerations of proceduralists who provide ESG and LSG

| Physiological eligibility criteria | Contraindications for ESG and LSG |
| --- | --- |
| - Excessive adiposity posing a long-term health risk indicated by a BMI in the obese I category (BMI ≥30kg/m^2^) if associated with a weight-related comorbidity at the time of the proceduralist appointment, or - Excessive adiposity posing a long-term health risk indicated by a BMI in the obese ≥II category (e.g. ≥35kg/m^2^) with or without an existing comorbidity such as hypertension, type 2 diabetes mellitus or cardiovascular disease. | - Liver cirrhosis particularly if associated with portal hypertension, coagulopathy or thrombocytopaenia. - A medical comorbidity not allowing general anaesthesia. - Current pregnancy or planned pregnancy within 12-18 months. Future pregnancy would need to be discussed on a case by case basis as no guidelines for bariatric endoscopic procedures exist. The Obesity Society (TOS) and American Society for Metabolic and Bariatric Surgery (ASMBS) recommend avoiding pregnancy for 12 to 18 months following a bariatric surgery (1). - Psychiatric disorders that would interfere with weight loss or lead to patient safety concerns. - Anatomic variation precluding safe progression to ESG. - Mucosal bleeding lesions such as ulceration. - Neoplastic or pre-malignant lesions. - Large hiatal hernias (>3cm). - Evidence of portal hypertension such as endoluminal varices or portal hypertensive gastropathy. |

# Usual care

### *Procedures*

The ESG was performed by one of the study site proceduralists (surgeon or gastroenterologist) trained in ESG using the Overstitch device (Apollo Endosurgery, Austin, TX). The LSG was performed as per standard practice by one of the study site surgeons. All patients were admitted as inpatients following their procedure at one of several available private hospital in Queensland, dependent on the availability of the proceduralist and geographical location of the patient.

### *Preoperative multidisciplinary intervention and follow-up: standard care*

Prior to the procedure, all participants attended outpatient consultations with a nurse and the proceduralist. Patients had two appointments with an Accredited Practising Dietitian (APD; Australian registered) to conduct a dietary assessment and to implement a very low calorie diet (VLCD) which was triaged according to BMI due to its association with decreased procedure-related complications (2). A BMI <35kg/m^2^ had 1-week VLCD, BMI 35-49kg/m^2^ had 2-weeks VLCD, and BMI >50kg/m^2^ had 3-weeks VLCD. The VLCD provided at least the minimum requirement for protein, fat, vitamins and minerals but limited carbohydrate. The VLCD limited total daily energy intake to less than 800 calories (3350kJ), thus inducing a state of mild ketosis (3). The VLCD was implemented using commercially available standardised meal replacements. A daily multivitamin, very low to no-energy beverages (i.e., water, soda water, tea and coffee, and artificially sweetened beverages), and “allowed” low-energy foods (a range of non-starchy vegetables, soups, sauces and condiments, herbs and spices) were also consumed. If a patient took hypoglycaemic medications and/or insulin, their medications were decreased during the VLCD. If preoperative nutrient deficiencies were identified via pathology, additional oral nutrient supplements were recommended on a case-by-case basis.

### *Postoperative multidisciplinary intervention and follow-up: standard care*

Following the procedure, a 6-week texture-modified post-procedure diet was implemented for both the ESG and LSG patients (Table S2), and dietary advice was given to maximise nutritional status. All patients were recommended to consume a daily multivitamin for life. Following the procedure, multidisciplinary follow-up was provided to all participants. Within two-weeks postoperatively, patients had at least one appointment with a registered nurse and an APD; appointments were all provided via telephone. All patients were prescribed a proton pump inhibitor for the first month postoperatively.

# Table S2: Post-procedure texture modified diet plan for ESG and LSG patients

| **Diet type and purpose** | **Description** | **Implementation** |
| --- | --- | --- |
| Clear fluids: aims to replace or maintain the hydration and allow for minimum residue in the intestinal tract. Nutritionally inadequate. | Composition: Only fluids or foods that are liquid at room temperature^a^. Typically includes fat-free clear soups, juice, jelly, cordial, and water. Black tea and coffee are usually excluded.  Amount: Sipping throughout the day, with a goal of 2.5L/day | During hospital admission (variable length)^b^ |
| Full fluids: aims to provide oral liquids to maintain hydration, reduce stomach distension, and leave minimum residue in the intestinal tract post-procedure. Nutritionally inadequate. | Composition: Only fluids or foods that are liquid at room temperature^a^. Typically includes fat-free clear soups, juice, milk, water, cordial, jelly, smoothies, high-protein oral nutrition supplement. Black tea, coffee and carbonated drinks are excluded.  Amount: Sipping throughout the day, with a goal of 2.5L/day | Day of discharge to day 13 post-procedure. |
| Full fluids plus puree foods: to provide food which is smooth, requires no chewing, and aims to be nutritionally adequate. No fluids consumed 30 minutes before or after food. | Composition: Commercial food which has the consistency of baby oatmeal or pudding; or home-made food which is pureed to a smooth consistency and can be easily cut with the side of a fork. Food should be cohesive enough to hold its shape on a spoon or if moulded.  Nutritional composition: goal of 50% protein, 30% low-carbohydrate vegetables, 20% wholegrains or legumes.  Amount: Commence with 4-5tsp of puree food per meal and aim for 0.5 per meal by day 27; with 5-6 meals per day. Goal of 2.5L fluid per day. | Day 14 to 27 post-procedure. |
| Soft foods: to provide food which can be easily chewed and nutritionally adequate, as a transition to a normal diet. No fluid-type restrictions, however avoid fluid consumed 30 minutes before or after food. | Composition: Includes foods which may be naturally soft or are cooked or altered to obtain a soft texture. Foods should be moist, easily crumbled, or served with sauce to increase the moisture content. Typically includes soft meat, fish, chicken, cereals, cooked vegetables, canned or fresh fruits. Skins, seeds, and bread are avoided.  Nutritional composition: goal of 50% protein, 30% low-carbohydrate vegetables, 20% wholegrains or legumes.  Amount: 0.5-1C per meal; with 5-6 meals per day. Goal of 2.5L fluid sipped throughout the day. | Day 28 to day 42 post-procedure. |
| Normal diet: to consume a wide variety of foods which are nutritionally adequate. No fluid-type restrictions. No fluid-type restrictions, however avoid fluid consumed 30 minutes before or after food. | Composition: Includes the full range of available foods, with guidance on food choice / meal plans to achieve a healthy dietary pattern.  Nutritional composition: goal of 50% protein, 30% low-carbohydrate vegetables, 20% wholegrains or legumes.  Amount: 0.5-1C per meal; with 5-6 meals per day. After 6 months post-procedure^c^, aim for 1C per main meal (3/day) and 0.5C per snack (2/day). Goal of 2.5L fluid sipped throughout the day. | Day 43 post-procedure onwards. |

C, cup; L, litre

1. Nutritional content of foods is modified based on patient symptoms. For example, if the patient is experiencing reflux, fat-free fluid options are used; if the patient is experiencing diarrhoea or dumping syndrome, sugar-free fluid options are used.
2. If patients have an unplanned extended length of stay in hospital progression to the next diet steps may occur during the hospital admission.
3. Patients may be able to achieve 1C portions of foods from variable timepoints, usually ranging from two weeks post-procedure to 18-months procedure.

**Table S3**: Characteristics of n=50 matched ESG and LSG patients according to procedure attendance.

| **Characteristics** | | **Attended the procedure (n=37)** | **Cancelled the procedure (n=13)** | **Group comparison** |
| --- | --- | --- | --- | --- |
| Age (y) | | 41.0 (9.8) | 43.9 (8.1) | *P*=.35 |
| Females, n (%) | | 31 (84) | 10 (77) | *P*=.68 |
| Ethnicity, n (%) | |  |  | *P*=.58 |
|  | Caucasian | 32 (86) | 13 (100) |  |
|  | Asian | 2 (5) | 0 |  |
|  | Black | 1 (3) | 0 |  |
|  | Indigenous Australian | 0 | 0 |  |
|  | Pacific Islander | 0 | 0 |  |
|  | Not disclosed | 2 (5) | 0 |  |
| Rural dwelling, n (%) | | 2 (5) | 1 (8) | *P*=.61 |
| SBP (mmHg) | | 123.5 (13.5) | 124.0 (9.7) | *P*=.91 |
| DBP (mmHg) | | 84.7 (10.0) | 79.9 (10.1) | *P*=.16 |
| T2DM, n (%) | | 1 (3) | 0 | *P*=.74 |
| HTN, n (%) | | 13 (35) | 2 (15) | *P*=.16 |
| Dyslipidaemia, n (%) | | 7 | 4 | *P*=.30 |
| OSA, n (%) | | 20 | 10 | *P*=.13 |
| OA/joint pain, n (%) | | 14 | 8 | *P*=.14 |
| NAFLD, n (%) | | 2 | 2 | *P*=.28 |
| PCOS ^e^, n (%) | | 4 | 1 | *P*=.83 |
| GORD, n (%) | | 12 | 8 | *P*=.07 |
| Depression or anxiety, n (%) | | 16 | 8 | *P*=.26 |
| GDM ^e^, n (%) | | 4 | 3 | *P*=.41 |
| IFG, n (%) | | 0 | 0 | N/A |
| Back pain, n (%) | | 23 | 9 | *P*=.46 |
| Asthma, n (%) | | 11 | 5 | *P*=.18 |

Data presented mean (standard deviation) unless otherwise specified. DBP, diastolic blood pressure; GDM, gestational diabetes mellitus; GORD, gastroesophageal reflux disease; HTN, hypertension; IFG, impaired fasting glucose; n, number of participants; NAFLD, non-alcoholic fatty liver disease; OA, osteoarthritis; OSA, obstructive sleep apnoea; PCOS, polycystic ovary syndrome; SBP, systolic blood pressure; T2DM, type II diabetes mellitus.

# References

1. Mechanick JI, Youdim A, Jones DB, Garvey WT, Hurley DL, McMahon MM, et al. Clinical practice guidelines for the perioperative nutritional, metabolic, and nonsurgical support of the bariatric surgery patient—2013 update: cosponsored by American Association of Clinical Endocrinologists, the Obesity Society, and American Society for Metabolic & Bariatric Surgery. Surg Obes Relat Dis. 2013;9(2):159-91.

2. Holderbaum M, Casagrande DS, Sussenbach S, Buss C. Effects of very low calorie diets on liver size and weight loss in the preoperative period of bariatric surgery: a systematic review. Surg Obes Relat Dis. 2017.

3. Tsai AG, Wadden TA. The evolution of very‐low‐calorie diets: an update and meta‐analysis. Obesity. 2006;14(8):1283-93.
